# Dr. David B. Horton

**Published:** 1906-08

**Authors:** 


### OBITUARY.

Dr. David B. Horton, of Wolcott, N. Y., a graduate of the University of Buffalo, 1874, died in his office July 9, 1906, from the effects of carbolic acid, presumably taken with suicidal intent. Recently he suffered a cerebral attack which was followed by despondency, this leading to the action above described. He had attained professional prominence in his time as president of the Medical Society of the County of Wayne.

